# Internal Drive or External Pull: Independent Innovation and Sustainable Growth of Chinese Agricultural Enterprises

**DOI:** 10.3390/foods13193185

**Published:** 2024-10-07

**Authors:** Lanlan Li, Qingning Lin, Xiudong Wang

**Affiliations:** Institute of Agricultural Economics and Development, Chinese Academy of Agricultural Sciences, Beijing 100081, China; 13831015012@163.com (L.L.); linqingning@caas.cn (Q.L.)

**Keywords:** independent innovation, food security, agricultural enterprises, sustainable growth, digital transformation

## Abstract

The independent innovation of agricultural enterprises ensures national food security through enhancing food production efficiency and optimizing food nutritional quality. Independent innovation is an important way for the sustainable growth of agricultural enterprises. However, in this process, there are issues such as long cycles and high risks. Based on this, using the panel data of China’s listed agricultural enterprises from 2007 to 2021, this study empirically examined the impact of independent innovation on the sustainable growth of agricultural enterprises, as well as the moderating effects of internal drivers (digital transformation) and external pull factors (government subsidies) through a two-way fixed effects model. The findings are the following: (1) The impact of independent innovation on the sustainable growth of agricultural enterprises exhibits an “inverted U-pattern”. When the R&D investment of agricultural enterprises accounts for 77.85% of operating income, the sustainable growth ability of agricultural enterprises is the highest. (2) Compared with large agricultural enterprises, small and medium-sized agricultural enterprises reach the threshold of independent innovation ability later, and the incentive effect range of independent innovation is longer. (3) Independent innovation exerts a more significant “inverted U” effect on the sustainable growth of non-state-owned agricultural enterprises and agricultural enterprises in the mature stage, while its impact on the sustainable growth of state-owned agricultural enterprises and agricultural enterprises in the growth and decline stages is not significant. (4) Government subsidies can help enhance the positive impact of independent innovation on the sustainable growth of agricultural enterprises when it does not exceed the threshold but cannot alleviate the negative impact of independent innovation on the sustainable growth of agricultural enterprises when it exceeds the threshold; investment in digital transformation not only helps to enhance the positive impact of independent innovation on the sustainable growth of agricultural enterprises when it does not exceed the threshold but also helps to alleviate the negative impact of independent innovation on the sustainable growth of agricultural enterprises when it exceeds the threshold. The research results provide data support for agricultural enterprises to carry out innovation activities under internal drive and external pull. At the same time, it is of great significance for the national implementation of the strategy of storing food in technology and food security strategy.

## 1. Introduction

In the context of globalization, sustainable development has increasingly become the focus of common concern for all countries in the world. From the perspective of the United Nations’ sustainable development goals [1,2] and planetary boundaries [3,4,5], sustainable development is essential. The goals of “climate action”, “underwater life”, and “terrestrial life” in the United Nations Sustainable Development Goals define the specific tasks of protecting the earth’s ecological environment, and the planetary boundaries also define the safe range of the impact of human activities on the earth’s ecosystem. Some studies have observed that the discourse of sustainability has evolved from focusing solely on the relationship between economic and environmental parameters to also encompassing social impacts [6]. However, in the process of globalization and technological updating, sustainable development goals are facing more and more challenges due to sudden factors such as the ongoing COVID-19 pandemic and trade wars. Globally, countries are increasing investment in scientific and technological innovation to promote the development of green technology, clean energy, circular economy, and other fields. Scientific and technological innovation can improve resource utilization efficiency, reduce environmental pollution, and provide technical support for achieving the goal of sustainable development [6,7]. Since the concept of sustainable development/sustainable growth was first introduced at the United Nations Conference on the Human Environment in 1972 [8], technological innovation has provided a strong impetus and numerous opportunities for sustainable development [9]. Consequently, independent innovation is regarded as a crucial strategic choice for nations aiming to enhance their sustainable development efforts.

Agriculture serves as the foundation of national and social development, and its stable growth is vital to national food security and strategic reserves [10]. Currently, China’s agriculture stands at a pivotal juncture in its transition to modern agriculture. Throughout the “14th Five-Year Plan” and beyond, agricultural enterprises in China are entrusted with numerous missions and responsibilities, including spearheading agricultural innovation, safeguarding the lifeblood of fundamental industries, and preserving national food security. Nevertheless, these enterprises continue to grapple with challenges such as the scarcity of leading technological enterprises and the absence of core technologies [11], which significantly hinder their sustainable growth. Consequently, promoting the sustainable growth of agricultural enterprises and cultivating a group of agricultural enterprises with strong independent innovation capabilities, outstanding economic performance, and a sense of social responsibility has become an urgent need to achieve the strategic goal of “keeping the rice bowl firmly in one’s own hands”. R&D investment is the starting point for independent innovation in agricultural enterprises and a key driving force for achieving sustainable development. According to relevant data, from 2015 to 2020, the average R&D investment scale of China’s agricultural enterprises increased from 59 million yuan to 95 million yuan, but it still remains less than half of that of listed agricultural enterprises on the A-share market (According to calculations by Wind Information Co., Ltd. (Shanghai, China), the average R&D investment scale of China’s A-share companies exceeded 250 million yuan in 2020. A-shares are ordinary stocks denominated in Chinese yuan, listed on the Shanghai and Shenzhen stock exchanges in China and traded in Chinese yuan. They constitute the primary market for domestic investors to engage in. Compared with non-listed agricultural enterprises, A-share agricultural enterprises possess significant advantages. In terms of data accessibility and research convenience, A-share agricultural enterprises exhibit relatively sufficient information disclosure, including company financial reports, company announcements, industry research reports, news media reports, etc. These pieces of information offer researchers a multidimensional perspective, aiding in a comprehensive understanding of the business situation and industry development trends of enterprises. In terms of policy and industry representativeness, A-share agricultural enterprises are often the key beneficiaries of national agricultural policies, with their development being directly influenced by policies. Meanwhile, A-share agricultural enterprises often occupy a leading position in the industry, thereby demonstrating their development strategies, business models, and technological innovations to a certain extent). In this way, under the background of increasing R&D investment of agricultural enterprises, can independent innovation lead to the sustainable growth of agricultural enterprises? Is there a crowding-out effect? In the process of independent innovation, should we rely on internal drive or external pull? Studying these issues holds significant importance for implementing the “storing grain in technology” strategy and realizing agricultural modernization.

The contribution of independent innovation to the economic performance of Chinese enterprises is increasingly evident, but it has not yet effectively supported the achievement of sustainable enterprise growth. The relevant literature on the relationship between independent innovation and enterprise development, both domestically and internationally, mainly focuses on the following aspects; the first view is the evaluation measures, evolution characteristics, and influencing factors of independent innovation [12,13]. Second, R&D investment, technological innovation, and enterprise development [14,15]. With the development of the theory of innovation ecosystems, enterprise innovation, as a systematic activity, places greater emphasis on the coordinated cooperation of multiple factors. The existing literature discusses the impact of independent innovation on the economic performance of enterprises, resulting in three primary arguments. One view is that independent innovation has a significant positive impact on enterprise growth. Chen et al. [16] and Han and Manry [17] found that enterprise R&D investment promotes the growth of enterprise value. Griliches [18] discovered through a study of 157 enterprises in the United States that innovation investment effectively enhances the market competitiveness of enterprises. Garner et al. [19] found that the efficiency of R&D investment significantly affects the innovation speed of enterprises, which further impacts their growth potential. Chung et al. [20] found that an increase in the intensity of enterprise R&D investment elevates the technological content of enterprises, thereby enabling them to generate excess returns. The second view is that independent innovation has a lag effect on the steady growth of enterprises. From the perspective of venture capital, He [21] found that R&D investment did not significantly increase the economic value of enterprises in the current period, confirming the lagged nature of the relationship. Wu et al. [22] and others conducted research on listed manufacturing companies and discovered that R&D investment in the current period, lag 1 and lag 2, all have a threshold effect on enterprise value. The third perspective is that independent innovation exhibits a nonlinear impact on the high-quality development of enterprises. Sun and Chen [23] discovered a significant “U-shaped” relationship between the intensity of R&D investment and enterprise viability through their research on listed companies. Zhang and Peng [24] observed a nonlinear “S-shaped” relationship between innovation intensity and enterprise survival after analyzing Chinese industrial enterprises. Yang et al. [25] conducted a sample survey of high-tech and non-high-tech enterprises in Taiwan, revealing a three-stage S-shaped relationship between R&D investment and enterprise performance.

Overall, while much research has focused on the significance of independent innovation in the survival and development of enterprises, there remain some shortcomings. Firstly, it has focused solely on a particular aspect of a company’s growth, such as the economy, environment, or society, without integrating both economic and social aspects into a comprehensive framework. Based on previous theoretical research and empirical observations, we found that economic and social performance have a direct and critical impact on the survival and development of enterprises. Economic performance is directly related to the viability of agricultural enterprises, while social performance is crucial for agricultural enterprises to obtain local support and resources. Although previous studies have recognized that the development of enterprises should be sustainable, their research mainly focuses on the economic level while giving less consideration to social performance. Agriculture itself is a multifaceted industry. To enhance the sustainable development capability of agricultural enterprises, it is necessary to fully strengthen communication and cooperation with stakeholders such as farmers, suppliers, and consumers, as well as enhance social influence. Secondly, despite some literature exploring the inverted U-shaped relationship between independent innovation and the sustainable growth of enterprises, there is a clear lack of research on the moderating effect between the two. Few studies have specifically focused on moderating the inverted U-shaped effect of independent innovation. In the process of independent innovation in enterprises, it is necessary to fully utilize the role of government subsidies. Government subsidies can provide financial support for R&D activities, reduce innovation costs and risks, and motivate enterprises to increase innovation investment. At the same time, digital transformation has become an important trend in the development of enterprises today, which can enhance innovation efficiency and fuel innovation momentum. Digital transformation brings new opportunities for breakthrough innovation in enterprises [26]. Therefore, we integrate government subsidies and digital transformation into the same theoretical framework from the perspectives of internal “driving” and external “pulling” of agricultural enterprises and explore their moderating effects in the process of leading sustainable growth of agricultural enterprises via independent innovation.

Based on this, this study utilized data from Shanghai and Shenzhen A-share listed agricultural enterprises from 2007 to 2021 to construct a research framework of independent innovation—internal drive/external pull—sustainable growth of enterprises, empirically examined the effect of independent innovation in leading the sustainable growth of agricultural enterprises, and explored the moderating roles of digital transformation and government subsidies.

## 2. Theoretical Analysis and Research Hypotheses

### 2.1. Impact of Independent Innovation on the Sustainable Growth of Agricultural Enterprises

Independent innovation is an effective strategy for the sustainable growth of enterprises [27,28]. In the initial stage of independent innovation, the increasing intensity of R&D investment in agricultural enterprises is conducive to creating value [29] and promoting the sustainable growth of enterprises. Based on the theory of value creation [30], enterprise value is the guarantee for the healthy and stable development of enterprises and the source of enhancing their core competitiveness. Nowadays, the intense competition among enterprises in the agricultural market puts forward higher requirements for independent innovation: (1) Independent innovation can create more new products for agricultural enterprises, and innovative agricultural products can generate higher economic benefits for enterprises, thereby improving the profitability of agricultural enterprises. (2) Independent innovation is conducive to enhancing the financing capabilities of agricultural enterprises. When enterprises inform creditors of their intention to raise funds through independent innovation, it is easier to obtain financing, exerting the “financing signal effect”. Independent innovation can effectively leverage the financial leverage of agricultural enterprises. (3) Enhancing the capability of independent innovation is beneficial for improving enterprises’ utilization of information and technology [31] and promoting more significant business performance in agricultural enterprises. On the one hand, continuous R&D investment sends a signal to the outside world that agricultural enterprises value innovation. Agricultural enterprises earn a good reputation through the “innovation signal effect”, which helps to attract more external opportunities, such as the attention of local governments, respect from the industry, and optimism from investors, in addition to increasing the intensity of R&D investment can increase the knowledge resources and enhance the innovation capability of agricultural enterprises.

However, independent innovation should have a reasonable limit [32]. When it exceeds the threshold, independent innovation will inhibit the sustainable growth of agricultural enterprises in the later stage of R&D investment. First, excessive independent innovation will occupy the resources of agricultural enterprises, including tangible resources such as capital, human resources, and material resources, as well as intangible resources such as time and technology, which may cause a conflict between “conservative management” and “innovative development”. Secondly, the R&D investment activities of agricultural enterprises have certain risks, and the increase in the intensity of independent innovation will exponentially expand the risks of development and production in enterprises, increasing the uncertainty of agricultural enterprises’ operations. Moreover, over-reliance on independent innovation could be linked to the suboptimal efficiency of agricultural enterprises, and blindly persisting with independent innovation could potentially result in operational challenges for agricultural enterprises.

Based on the above analysis, the following research hypothesis is proposed:

**H1.**  *The impact of independent innovation on the sustainable growth of agricultural enterprises exhibits an ‘inverted U’ pattern.*

### 2.2. The Moderating Effect of Investment in Digital Transformation and Government Subsidies

In today’s digital age, digital transformation has become a trend, and the intensity of investment in digital transformation has a significant impact on the independent innovation and research and development activities of enterprises. At the same time, due to the inherent public welfare and social nature of independent innovation in agricultural enterprises, the difficulties and risks associated with it are more severe compared with other industries. Relying solely on the invisible hand to achieve regulation makes it difficult to attain Pareto optimality, thus necessitating the role of government intervention. Therefore, from the perspectives of internal drive and external pull, we incorporate digital transformation and government subsidies into the theoretical analysis framework of the impact of independent innovation on the sustainable growth of agricultural enterprises, exploring the moderating effects of digital transformation and government subsidies.

#### 2.2.1. The Moderating Role of the Intensity of Digital Transformation Investment between Independent Innovation and the Sustainable Development of Agricultural Enterprises

The increased investment in the digital transformation of agricultural enterprises entails the introduction, application, reform, and innovation of digital technology in these enterprises, as well as a heightened emphasis on digital transformation. The greater the investment in the digital transformation of agricultural enterprises, the more it facilitates the precise allocation of R&D capital and promotes the sustainable development of these enterprises. During the initial stages of independent innovation, when the intensity is relatively low, the investment in digital transformation promotes the positive impact of independent innovation on the sustainable development of agricultural enterprises. This is primarily reflected in three key areas: Firstly, the investment in digital transformation of agricultural enterprises can effectively stimulate leaps in R&D investment [33]. Secondly, the increased investment in the digital transformation of agricultural enterprises aids these enterprises in adopting more suitable business models, further fostering strategic innovation and, thus, significantly enhancing R&D investment [34]. Thirdly, the more advanced the digital infrastructure of agricultural enterprises, the more it supports these enterprises in enhancing dynamic capabilities, optimizing resource allocation, and improving R&D efficiency.

When independent innovation reaches a significant intensity in the mature stage, digital transformation investment can help mitigate the negative impact of independent innovation on the sustainable growth of agricultural enterprises. It is mainly reflected in three aspects: information processing, specialized division of labor, and risk avoidance. First, the digital resource platform and infrastructure formed in the early stage provide advanced processing means for the market information of R&D investment. The mature and perfect information receiving and processing system makes agricultural enterprises more precise in innovation investment, more targeted in determining the direction and focus of R&D, and increase the intensity and efficiency of R&D investment. Secondly, increasing investment in digital transformation can help agricultural enterprises break through traditional industry boundaries [35,36], promote the integration of the primary, secondary, and tertiary sectors in agricultural enterprises, boost industrial integration by opening up and improving the agricultural industrial chain, and help improve the level of professional division in R&D and production. Finally, increasing investment in digital transformation will help agricultural enterprises identify external opportunities, improve the ability to avoid external risks, reduce innovation costs, and ultimately promote the sustainable growth of agricultural enterprises.

Based on this analysis, this paper puts forward the following research hypotheses.

**H2a.**  *The intensity of digital transformation investment helps to strengthen the positive impact on the sustainable growth of agricultural enterprises when independent innovation does not exceed the threshold.*

**H2b.**  *The intensity of digital transformation investment can help mitigate the negative impact on the sustainable growth of agricultural enterprises after independent innovation exceeds the threshold.*

#### 2.2.2. The Moderating Role of Government Subsidies between Independent Innovation and Sustainable Development of Agricultural Enterprises

For agricultural enterprises, whether in agriculture, forestry, animal husbandry and fishery, agricultural and sideline food processing, food manufacturing, or refined tea manufacturing, they all have the characteristics of long investment periods, slow return on investment, and high susceptibility to uncertainties. At the same time, compared with the manufacturing and service industries, the capital held by agricultural enterprises themselves is not dominant, so they are more dependent on government subsidies, and their independent innovation is more responsive to government subsidies. In the initial stage of independent innovation, government subsidies will promote the incentive effect of independent innovation on the sustainable development of agricultural enterprises. Firstly, government subsidies can bring direct cash inflows to agricultural enterprises, thereby supporting their R&D investment [37], new variety cultivation, and new technology promotion activities; meanwhile, these subsidies differ from debt and equity financing in that they do not need to be repaid by enterprises, thus eliminating part of the financing pressure on agricultural enterprises. Secondly, government subsidies supporting technological innovation in agricultural enterprises will send signals to the outside world [38]. Innovative agricultural enterprises receiving government subsidies indicate that they possess strong technological innovation capabilities and innovative projects. Based on the signal effect of government subsidies, social resources such as R&D talents and partners will also be gathered for agricultural enterprises.

However, when the intensity of independent innovation in agricultural enterprises exceeds the threshold value, government subsidies may aggravate the negative impact of independent innovation on the sustainable growth of agricultural enterprises. First, when the intensity of independent innovation in enterprises exceeds the threshold value, agricultural enterprises may fall into the misconception of resource mismatch [39]. At this time, the addition of government subsidies may lead to more resources being invested in these excessive innovation activities while neglecting other more beneficial and sustainable business areas of the enterprises, resulting in an unreasonable allocation of resources. Secondly, excessive independent innovation may reduce the innovation efficiency of agricultural enterprises. In this case, government subsidies may further encourage disorganized innovation in agricultural enterprises, causing them to spread their efforts across numerous innovation projects instead of focusing on overcoming key technologies or launching products with market competitiveness. Third, when the intensity of independent innovation exceeds a reasonable range, the market may question the innovation achievements of agricultural enterprises. At this time, government subsidies may be misinterpreted by the market as indicating that the enterprises rely on external support rather than their own strength, thereby reducing market confidence in agricultural enterprises and affecting their sustainable growth.

Based on the above analysis, this paper puts forward the following research hypotheses.

**H3a.**  *Government subsidies positively moderate the positive impact of independent innovation on the sustainable growth of agricultural enterprises when it does not exceed the threshold.*

**H3b.**  *Government subsidies cannot alleviate the negative impact of independent innovation on the sustainable growth of agricultural enterprises once it exceeds the threshold.*

**Figure 1:**
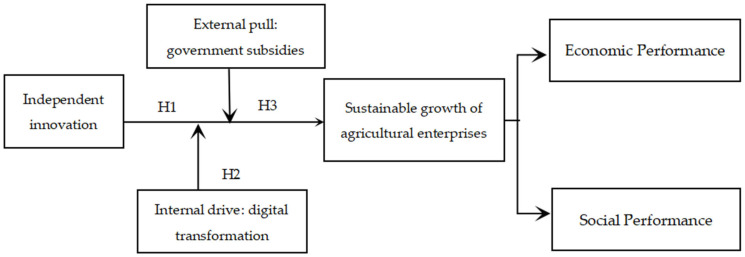
The impact mechanism of independent innovation on the sustainable growth of agricultural enterprises.

Based on the above theoretical analysis, the research framework of this paper is shown in Figure 1.

## 3. Materials and Methods

### 3.1. Sample Selection and Data Collection

Based on the perspective of the whole agricultural industry chain and referring to “the concept of diversified food”, listed agricultural enterprises include agricultural, forestry, animal husbandry, and fishery service listed companies in the primary industry, narrowly defined, food processing, beverage manufacturing, wood processing industries in the secondary industry, and agricultural products circulation, rural finance, and other agricultural, forestry, animal husbandry, and fishery service listed companies in the tertiary industry. As the number of listed agricultural enterprises in the tertiary industry is very small and unrepresentative, this paper selected agricultural enterprises in the primary and secondary industries as research samples according to the industry classification results of listed companies by the CSRC (The China Securities Regulatory Commission (CSRC) [Beijing, China], in accordance with laws and regulations, uniformly supervises and manages the national securities and futures market, maintains the order of the securities and futures market and ensures its lawful operation. The CSRC conducts industry classification and other regulatory work on listed companies, providing an important guarantee for the healthy development of the capital market) in the third quarter of 2019; at the same time, considering the rationality and reliability of the research indicators, ST* and ST (“ST” is the abbreviation for “Special Treatment”. When a listed company experiences abnormal financial or other conditions that put its shares at risk of delisting, the exchange will impose special treatment on its stock trading. “ST*” generally refers to companies under special treatment which face higher risks. The stocks of companies under special treatment are usually marked with the prefix “ST” or “ST*” and may have different price limits compared with normal stocks. These companies often have poor performance and significant operational uncertainties), companies with poor performance and agricultural listed enterprises with abnormal indicators were excluded.

### 3.2. Variable Measurement

#### 3.2.1. Dependent Variable

Sustainable Growth of Agricultural Enterprises (SGAEs). The sustainable growth of agricultural enterprises is characterized by sustainability, dynamism, profitability, volatility, and expansion [40], and its level is influenced by many factors. Firstly, agricultural enterprises carrying out innovation possess public, fundamental, and social characteristics. They should not only focus on improving operational income and other economic benefits but also shoulder social responsibilities such as ensuring food security and promoting rural economic development. Therefore, when measuring the sustainable growth indicators of agricultural enterprises, it is necessary to take into account economic quality, growth rate, and social performance. Secondly, unlike high-polluting industries and heavy industrial enterprises, agricultural enterprises have very low carbon emissions. There are significant differences between agricultural enterprises and traditional high-carbon-emission industries such as steel and cement, and agricultural enterprises show a relatively low trend of carbon emissions [41,42]. According to the “Comprehensive List of Environmental Protection (2021 Edition)” [43] issued by the Ministry of Ecology and Environment (Beijing, China), there are few agriculture-related industries in high-pollution and high-environmental-risk sectors. Therefore, based on the concept of sustainable development and growth of agricultural enterprises, this paper drew on the research of Ruf et al. [44], Freedman and Jaggi [45], Husted et al. [46], and Trebucq and d’Arcimoles [47] and combines it with the actual development situation of agricultural enterprises in China. It establishes an evaluation index system for the sustainable growth of agricultural enterprises from the five dimensions of profitability, scale capacity, debt-paying ability, growth potential, and social performance.

The sustainable growth of agricultural enterprises must prioritize profitability, making it a key factor in assessing their long-term success. Profitability reflects the ability of assets to generate income, the profit level from sales, and the return on invested capital. Referring to the research of Han and Fan [48], we measured the profitability of enterprises by the rate of return on total assets, operating gross profit margin, net profit margin on sales, and return on invested capital. In addition to profitability, sustainable growth often requires expanding the enterprise’s scale, which is evident in the growth of fixed assets and operating income. Referring to the research by Han and Fan and Xu et al. [48,49], we measured the scale capacity of enterprises by the scale of fixed assets and operating revenue scale. Solvency is also critical; while financial leverage can lower capital costs, it also introduces debt repayment obligations. Thus, the capital structure acts as a “double-edged sword,” influencing long-term sustainability. Referring to the research of Xiao et al. [40], we measured the solvency by quick ratio and current ratio. The enterprise’s growth capacity, which reflects the speed of development, is another essential measure. By balancing growth quality with speed, agricultural enterprises can enhance their market competitiveness. Referring to the research by Xiao et al. and Zhai and Fang [40,50], we measured the growth capacity by the growth rate of total assets and the growth rate of operating revenue. The growth rate of total assets reveals the company’s development speed, while the growth rate of operating revenue signifies an increase in market share. Moreover, social performance plays a significant role in indicating the sustainable growth capability of agricultural enterprises. Referring to the research of Han and Fan, Wang and Bao and Wen and Fang [48,51,52], we measured social performance through social donation and external guarantee. Agricultural enterprises often show heterogeneity in their social donations, which can include materials such as seeds, grains, and agricultural products that are directly used in agricultural production. These contributions help improve the conditions of agricultural production, enhance the yield and quality of agricultural products, and promote the development of the rural economy and the livelihoods of farmers. Additionally, external guarantees provided by agricultural enterprises also contribute to their overall social responsibility and sustainable growth.

Drawing on the research on enterprise growth [53,54], we employed the principal component analysis method to extract the principal components from various indicators within the dimension of sustainable growth (Table 1). Based on the selected principal components, we established a component analysis model and calculated the scores of the principal components as a metric for assessing the sustainable growth capability of agricultural enterprises.

#### 3.2.2. Core Explanatory Variable

This paper employed R&D investment as a proxy variable for enterprise-independent innovation. R&D investment serves as the fundamental resource for enterprises to conduct innovative activities. A higher level of R&D investment typically indicates a greater commitment and intensity to independent innovation. The current literature on measuring R&D investment primarily distinguishes between two approaches, “absolute and relative values”. Considering that R&D intensity can be used for comparative analysis among agricultural enterprises and drawing on the practices from existing literature [55,56,57], this paper selected the ratio of R&D investment to operating revenue to measure independent innovation.

#### 3.2.3. Moderating Variables

According to existing research on digital transformation [58,59,60,61,62,63], there are three main methods for measuring the investment intensity variables of digital transformation: (1) Employ a ‘0–1’ dummy variable to denote whether digitization has been implemented. (2) Digitalization is measured by analyzing the frequency of keywords related to digitization in enterprise annual reports. (3) Digital economic indicators at the city level are utilized to assess the level of digitization. However, the dummy variable setting fails to reflect the scale of an enterprise’s digital resources. Moreover, many depictions of digitization in enterprise annual reports serve merely as background introductions, indicating a lower level of actual digitization. This, therefore, fails to reflect the true extent of an enterprise’s digitization efforts. There is a significant discrepancy between the macro perspective of digitization and the actual level of enterprise digitization. Therefore, by taking into account the aforementioned factors and drawing on the practices described in the existing literature [64], digital resources encompassing intangible assets were defined to include software, data resources, electronic platforms, information systems, and intelligent technology resources. Similarly, the digital resources pertaining to fixed assets comprise computer equipment, communication devices, and electronic equipment. The indicators obtained by screening and aggregating the digital transformation investment resources within enterprise assets serve as a proxy for the intensity of an enterprise’s digital transformation investments.

In the current study, the measurement methods for moderating variables of government subsidies mainly included taking the logarithm of government subsidies, the ratio of government subsidies to total assets, and the ratio of government subsidies to operating income [37]. Considering that the absolute value of government subsidies cannot reflect the intensity of subsidies, this study used the ratio of government subsidies to operating income to measure the intensity of government subsidies and employed the ratio of government subsidies to total assets for robustness tests.

#### 3.2.4. Control Variables

Drawing on existing research [28,65,66,67,68], and to avoid overlap with the financial indicators that are the explained variables, this study selected representative control variables: the total assets of the enterprise, represented by asset size, measured by taking the logarithm of the total assets; the P/E ratio, characterized by market estimates; the total asset turnover rate, an indicator of operational performance. Among these, a larger asset size indicates a stronger overall corporate strength, which is more conducive to the sustainable growth of the enterprise. The P/E ratio reflects investors’ expectations of a company’s growth prospects and is a reflection of the market’s optimistic view of the company’s growth prospects. The higher the P/E ratio, the greater the likelihood of the company’s sustainable growth. The total asset turnover rate reflects a company’s operational capacity. The higher the total asset turnover rate, the more conducive it is to the sustainable growth of the enterprise.

### 3.3. Descriptive Statistics

Table 2 presents the descriptive statistical results of the main variables. From the perspective of the sustainable growth score values of agricultural enterprises, the highest score value was 9.760, and the lowest was −9.990. There was a significant difference between the maximum and minimum values. During the sample period, there were significant differences in the sustainable growth capabilities of agricultural enterprises. The average and median proportions of R&D investment in agricultural enterprises in operating income were 1% and 0.65%, respectively, with a standard deviation of 0.01. In terms of the investment intensity in digital transformation, the overall investment intensity in digital transformation of China’s agricultural enterprises is on the rise, but nearly 30% of agricultural enterprises have little or no investment in digital resources. After taking the logarithm of the digitalization investment, there was a significant difference between the maximum value of 19.41 and the minimum value of −1.715, reflecting the uneven development of the investment intensity in digital transformation among agricultural enterprises in China. From the perspective of government subsidies, the average proportion of government subsidies in operating income in China stood at 0.7%, with a maximum of 16.2% and a minimum of 0, indicating that there are obvious differences in government subsidies enjoyed by different agricultural enterprises. Regarding the control variables, the average enterprise size was 21.86, the average p/E ratio was 0.79, and the average total asset turnover rate was 0.81.

### 3.4. Empirical Model

To test the above assumptions, the following benchmark models were constructed.

(1) $${SGAE}_{i,t}=\alpha_{0}+\alpha_{1}R\&D_{i,t}+\alpha_{2}R\&{D_{i,t}}^{2}+\alpha_{3}\sum_{j}Controls_{i,t}+\eta_{t}+\eta_{ind}+\varepsilon_{i,t}$$

(2) $$\begin{array}{l} {SGAE}_{i,t}=\gamma_{0}+\gamma_{1}R\&D_{i,t}+\gamma_{2}R\&{D_{i,t}}^{2}+\gamma_{3}R\&D_{i,t}\times \ln{Dig}_{i,t}+\gamma_{4}R\&{D_{i,t}}^{2}\\ \times \ln{Dig}_{i,t}+\gamma_{5}\ln{Dig}_{i,t}+\gamma_{6}\sum_{j}{Controls}_{i,t}+\eta_{t}+\eta_{ind}+\varepsilon_{i,t} \end{array}$$

(3) $$\begin{array}{l} {SGAE}_{i,t}=\beta_{0}+\beta_{1}R\&D_{i,t}+\beta_{2}R\&{D_{i,t}}^{2}+\beta_{3}R\&D_{i,t}\times {Sub}_{i,t}\\ +\;\beta_{4}R\&{D_{i,t}}^{2}\times {Sub}_{i,t}+\beta_{5}{Sub}_{i,t}+\beta_{6}\sum_{j}{Controls}_{i,t}+\eta_{t}+\eta_{ind}+\varepsilon_{i,t} \end{array}$$

To investigate the impact of independent innovation on the sustainable growth of agricultural enterprises, we have constructed a benchmark regression model (1). Models (2) and (3) were utilized to examine the moderating effects of the intensity of digital transformation investment and government subsidies, with a focus on the coefficients of R&D_i,t_*lnDig_i,t_, *R&D_i,t_*^2^*lnDig_i,t_, R&D_i,t_*Sub_i,t_, and *R&D_i,t_*^2^*Sub_i,t_.

Where *SGAE_i,t_* indicates the sustainable growth ability of agricultural enterprises; *R&D_i,t_* is the proportion of *R&D_i,t_* investment in operating income, representing the independent innovation ability of enterprises; *lnDig_i,t_* stands for the investment intensity of enterprises’ digital transformation; *Sub_i,t_* represents government subsidies; *Controls_i,t_* is the control variables affecting the value of agricultural enterprises; *η_t_* denotes the time fixed effect; *η_ind_* denotes the industry fixed effect; and *ε_i,t_* is the random disturbance term.

## 4. Results

### 4.1. Analysis of Benchmark Regression Results

Table 3 presents the regression results of the impact of independent innovation on the sustainable growth of agricultural enterprises. As shown in column (1), after adding industry-fixed effects and time-fixed effects to the model, the regression coefficient of independent innovation on the sustainable growth of agricultural enterprises is significantly positive, indicating that an increase in independent innovation promotes the sustainable growth of agricultural enterprises, and the “value-added” effect of independent innovation on agricultural enterprises is evident. The impact of control variables on the sustainable growth of agricultural enterprises is shown in column (2). Enterprise size and total asset turnover have a significant positive impact on the sustainable growth of agricultural enterprises, while P/E has a negative impact on the sustainable growth of enterprises. After controlling for all control variables, the test results are shown in column (3). In order to avoid endogenous problems, column (4) uses the lagged term of independent innovation as an instrumental variable and employs the two-stage least squares method (2SLS) for regression analysis. Additionally, robust standard error estimation is used, and the regression results are essentially consistent with those in column (3). Furthermore, the Cragg-Donald Wald F statistic value for the impact of independent innovation on the sustainable growth of agricultural enterprises was 5957.870, which is significantly higher than the 10% maximal IV size value of 16.38 proposed by Stock and Yogo [69] and passed the test for weak instrumental variables.

Refer to the U-shaped (or inverted U-shaped) curve test method proposed by Haans et al. [70] to determine whether the regression results meet the three conditions of the U-shaped (or inverted U-shaped) curve. First of all, according to the results in column (5) of Table 3, the primary term coefficient of independent innovation was significantly positive (1.504), and the secondary term coefficient was significantly negative (−0.966), which meets the first condition of inverted U-shaped curve judgment. Secondly, after calculation, the value range of the intensity of independent innovation was [0.1, 1], the left-end slope K1 was 1.3108, and the right-end slope K2 was −0.428, which meets the second condition for judging the inverted U-shaped curve. Finally, the threshold value of the inverted U-shaped curve of 0.778 was within the value range of the intensity of independent innovation [0.1, 1], which meets the third condition required by the judgment of the inverted U-shaped curve, indicating that there is a significant inverted U-shaped relationship between independent innovation and the sustainable growth of agricultural enterprises. Hypothesis H1 was verified. After calculation, when the R&D investment of agricultural enterprises accounted for 77.85% of the operating income, the effect of promoting the sustainable growth of agricultural enterprises was most pronounced. As of 2021, 2% of Chinese agricultural enterprises have just reached the optimal threshold for independent innovation, 2% are about to reach it, 8% have exceeded it, and 88% have not yet reached it.

In order to ensure the integrity and accuracy of the research results, column (6) considers the endogenous nature of independent innovation, takes the coefficient of the quadratic term of the lagged term of independent innovation as the instrumental variable, and employs the two-stage least squares method (2SLS) for regression analysis. The coefficient of the quadratic term of independent innovation was significantly negative, and the Cragg-Donald Wald F statistic value was 309.401, which is significantly higher than the 10% maximal IV size value of 16.38 proposed by Stock and Yogo [69]; it passed the weak instrumental variable test.

### 4.2. Robustness Tests

By conducting robustness tests, we further verified the reliability and stability of the research findings. (1) Replacement of Dependent Variables. Drawing on the existing literature [64], the academic community typically measures enterprise value using the relative ratio of net profit to total profit as an indicator. In this study, return on equity (ROE), net profit on total assets (NPOA), and return on total assets (ROA) were used to replace the dependent variable, agricultural enterprise sustainable growth ability (SGAE). The results are shown in Table 4 (1), (2), (3). The relationship between independent innovation and the three indicators exhibits an inverted U-shaped pattern, confirming the robustness of the regression results in this paper.

(2) Change of Sample Period. To avoid the impact of the 2008 financial crisis, this paper shortens the time window and conducts a robustness analysis based on samples from 2010 to 2021. The test results are shown in column (4) of Table 4. There is a significant inverted U-shaped relationship between independent innovation and the sustainable growth of agricultural enterprises, which demonstrates the robustness of the results in this paper.

(3) Exclude samples that have not invested in digital transformation. Although digital transformation investment in agricultural enterprises has become an inevitable trend, there are still some agricultural enterprises that lack investment in digital resources or have not even initiated the process of digital transformation. Therefore, enterprises that have not invested in digital resources were excluded from the robustness analysis. The regression results are presented in column (5) of Table 4. The results show that there is a significant inverted U-shaped relationship between independent innovation and the sustainable growth of agricultural enterprises, indicating the robustness of the regression results in this paper.

(4) Substituting the core explanatory variables. This study used the proportion of R&D investment in total assets to substitute the core explanatory variables for a robustness test. The results in column (6) of Table 4 show that the relationship between independent innovation and the sustainable growth of agricultural enterprises remains inverted U-shaped.

### 4.3. Heterogeneity Analysis

#### 4.3.1. Nature of Property Rights

The management systems of state-owned enterprises are relatively conservative, and due to the fact that they can usually obtain governmental shielding and easy access to policy subsidies [71,72], the enthusiasm for independent innovation is low. Compared with state-owned agricultural enterprises, non-state-owned agricultural enterprises have greater incentive and impetus for independent innovation in order to pursue sustainable growth in the fierce market competition. At the same time, the liquidity of private agricultural enterprises and foreign-funded agricultural enterprises is relatively high, and the cross-disciplinary flow of knowledge is frequent. Therefore, the impact of independent innovation on the sustainable growth of agricultural enterprises may vary due to differences in the nature of property rights.

To verify whether there is heterogeneity in the impact of independent innovation on the sustainable growth of agricultural enterprises based on property rights, dummy variables are created based on the property rights nature, with state-owned enterprises coded as 1 and non-state-owned enterprises as 0. Table 5’s regression analysis results indicate that the primary and secondary coefficients of independent innovation for the non-state-owned agricultural enterprise group were significant, aligning with the characteristics of an “inverted U” relationship, while the coefficient estimation results for the state-owned agricultural enterprise group failed to pass the significance test. This suggests that, compared with state-owned agricultural enterprises, independent innovation plays a more significant role in the sustainable growth of non-state-owned agricultural enterprises. To further validate the robustness of the inter-group differences, a Chow test was conducted. The empirical results showed that the differences between the two groups passed the significance test.

#### 4.3.2. Enterprise Scale

Similar to the heterogeneity of light and heavy assets, large enterprises may also encounter the challenge of “being too big to turn around” [73]), which leads to agricultural enterprises being hesitant to invest in risky R&D endeavors. However, listed small and medium-sized agricultural enterprises possess considerable strength and higher flexibility in operation and management [74], enabling them to adjust their asset allocation more swiftly. Even if independent innovation decisions turn out to be suboptimal, they are less likely to be locked into previous mistakes. Therefore, independent innovation may have a higher efficiency in influencing the sustainable growth of agricultural enterprises.

To verify whether there is heterogeneity in the relationship between independent innovation and sustainable growth of agricultural enterprises across different enterprise sizes, dummy variables were set based on the enterprise scale and divided according to the median total asset size of the sample agricultural enterprises. Those above the median were classified as large, while those below were small- to medium-sized. As shown in Table 6, for both large and small to medium-sized agricultural enterprises, the relationship between independent innovation and the sustainable growth of agricultural enterprises exhibited an inverted U-shaped pattern. After calculation, the threshold values for large agricultural enterprises and small- to medium-sized agricultural enterprises were 52.12% and 96.64%, respectively. This indicates that large agricultural enterprises are the first to reach the independent innovation threshold, and once the independent innovation intensity reaches 52.12%, it begins to inhibit the sustainable growth of the enterprises. On the other hand, the positive effect of independent innovation on the sustainable growth of enterprises is longer for small and medium-sized agricultural enterprises. Only when the independent innovation intensity reaches 96.64% might it begin to hinder the sustainable growth of the enterprises.

#### 4.3.3. Enterprise Lifecycle

An enterprise is an organization with a life cycle. During different stages of the cycle, enterprises exhibit variations in scale, profitability, and willingness to invest in R&D, with corresponding shifts in the key constraints affecting enterprise value [75]. In the growth stage, agricultural enterprises face tight financing constraints and high capital expenditures [76], leading to insufficient independent innovation experience and low innovation willingness. Furthermore, the success rate of independent innovation is low due to a lack of capital accumulation and experienced R&D personnel. However, after a period of accumulation, agricultural enterprises in the mature stage have become increasingly sophisticated in terms of production models, organizational structures, and sales networks. Thanks to their relatively stable profits during this stage, coupled with early market exploration and independent innovation experience summaries, these enterprises find it easier to target their independent innovation toward sustainable growth opportunities, often ensuring that their independent innovation is “well-targeted” [77]. As agricultural enterprises enter the recession stage, the value-added effect of independent innovation on their sustainable growth diminishes due to a loss of profit growth opportunities, worsening financial conditions, difficulties in raising funds, managerial irresponsibility, and a lack of attention or awareness toward independent innovation.

To verify whether independent innovation in different life cycles of enterprises has a heterogeneous effect on the sustainable growth of agricultural enterprises, dummy variables are set according to the enterprise life cycle. Drawing on the practices of existing scholars [78], the life cycle of agricultural enterprises was divided into three stages: growth stage, maturity stage, and decline stage. The specific criteria for classification are shown in Table 7. As can be seen from Table 8, the relationship between independent innovation in mature agricultural enterprises and their sustainable growth clearly conforms to the characteristics of an “inverted U-shaped” relationship, while the impact of independent innovation in growth and decline stages on the sustainable growth of agricultural enterprises is not significant. This indicates that, compared with agricultural enterprises in the growth and decline stages, those in the maturity stage have relatively rich independent innovation experience and can leverage the advantages of specific resources, resulting in a more pronounced promoting effect of their independent innovation on sustainable growth.

## 5. Further Analysis: The Moderating Effect of Digital Transformation and Government Subsidies

Column (1) and column (2) in Table 9 show the impact of independent innovation on the sustainable growth of agricultural enterprises after incorporating the moderating variable of digital transformation investment intensity. According to column (1) and column (2), the coefficient of the interaction term between independent innovation itself and digital transformation investment intensity was significantly positive, as well as the coefficient of the interaction term between the square of independent innovation and digital transformation investment intensity. Without the moderating effect, the slope of self-innovation after crossing the threshold value for the sustainable growth of agricultural enterprises was 7.624. However, when the moderating variable of digital transformation investment intensity was included, the slope after self-innovation crossed the threshold became −3.934, with the absolute value of the slope decreasing. This indicates that, with the inclusion of the moderating variable of digital transformation investment intensity, the slope of the inverted U-shaped curve for the sustainable growth of agricultural enterprises after self-innovation exceeds the threshold becomes gentler. Combining the results of models (1) and (2), research hypothesis H2 was supported; that is, when the intensity of self-innovation does not exceed the threshold value, digital transformation investment intensity enhances the positive impact of self-innovation on the sustainable growth of agricultural enterprises. When self-innovation exceeds the threshold, digital transformation investment can effectively mitigate the negative impact of self-innovation on the sustainable growth of agricultural enterprises.

Column (3) and column (4) in Table 9 show the impact of independent innovation on the sustainable growth of agricultural enterprises after incorporating the adjustment item of government subsidies. According to column (3), the coefficient of the interaction between endogenous innovation and government subsidies was significantly positive. From column (4), it can be seen that the coefficient for the interaction between the square of independent innovation and government subsidies was significantly negative. Without the moderating effect, the slope of endogenous innovation beyond the threshold to the sustainable growth of agricultural companies was calculated to be −0.008. When the government subsidy adjustment was added, the slope of endogenous innovation beyond the threshold was calculated to be −0.658, and the slope became steeper. This indicates that after adding the government subsidy adjustment, the slope of the inverted U-shaped curve for the sustainable growth of agricultural companies becomes steeper when endogenous innovation exceeds the threshold. Based on the combined results of models (3) and (4), research hypothesis H3 was verified; that is, when the intensity of endogenous innovation does not exceed the threshold, government subsidies strengthen the positive impact of endogenous innovation on the sustainable growth of agricultural companies. Once independent innovation surpasses the threshold, government subsidies cannot alleviate the negative impact of independent innovation on the sustainable growth of agricultural enterprises.

**Figure 2:**
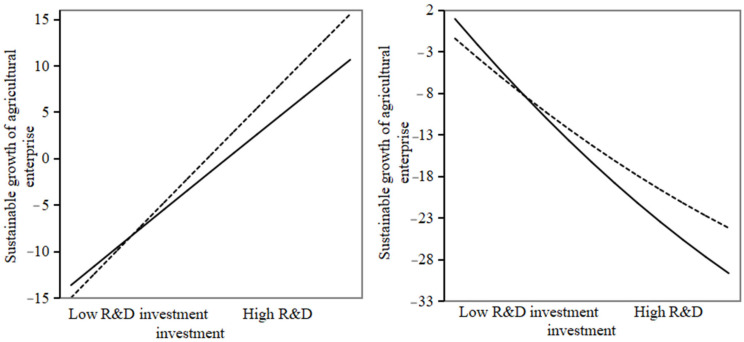
Moderating effect of investment intensity in digital transformation. Note: The solid line in the figure indicates low digital transformation investment, and the dotted line indicates high digital transformation investment.

Figure 2 illustrates the moderating effect of the intensity of investment in digital transformation. In the initial stages of agro-industry innovation, investment in digital transformation helps to enhance the precision of agro-industry enterprises’ innovation investment, and by enhancing digital infrastructure, fostering R&D collaboration, and promoting knowledge sharing, thereby strengthening the incentive effect of agro-industry innovation on sustainable growth. In the later stages of increased agro-industry innovation intensity, investment in digital transformation can alleviate the negative impact of agro-industry innovation on sustainable growth by enhancing efficiency monitoring and risk mitigation. For example, for seed enterprises with a high degree of digital transformation, using digital technology in breeding research and development to conduct multi-omics research combining genomic and phenotypic data is conducive to promoting intelligent design breeding, which greatly improves the breeding efficiency and economic value of the enterprise.

**Figure 3:**
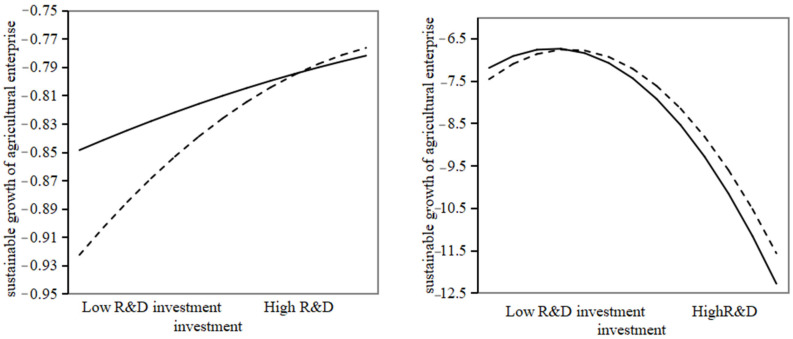
Moderating effect of government subsidies. Note: The solid line in the figure indicates low government subsidies, and the dotted line indicates high government subsidies.

Figure 3 illustrates the moderating effect of government subsidies. In the initial stages of independent innovation, government subsidies can provide additional financial support to enterprises, alleviate their financing constraints, and enable smoother innovation processes. Moreover, the government uses subsidy policies to guide the innovation direction of agricultural enterprises, aligning them more closely with the national development strategy and industrial policies. In the later stages, as the intensity of independent innovation increases, excessive government subsidies can crowd out private investment. This is because enterprises may view government subsidies as a cheaper source of funds, thereby reducing their reliance on their own funds or other financing channels. This could lead to a decline in investment efficiency in agricultural enterprises, affecting their long-term development. Additionally, government subsidies can distort market competition, giving subsidized agricultural enterprises an unfair competitive advantage in the market, which hinders their sustainable growth. Furthermore, government subsidies foster rent-seeking behavior in agricultural enterprises. According to the data from the 2021 Report on Innovation in China’s Agricultural Enterprises, the current proportion of listed agricultural enterprises in China’s listed enterprises is not high, and the proportion of innovative ones is even lower. The proportion of listed agricultural enterprises in China has dropped from 11.5% to 9.5%. In many regions across the country, the R&D environment, infrastructure, and intellectual property protection for agricultural enterprise innovation are far from perfect. To encourage agricultural enterprises to increase innovation, the government has implemented a series of subsidy policies. Many agricultural enterprises, in a bid to seize the business opportunities presented by this policy dividend, have diverted their efforts toward producing and selling government subsidy list projects rather than relying on technological innovation to develop products, failing to play a role in promoting the sustainable growth of agricultural enterprises.

## 6. Discussion

To deeply explore the effects and mechanisms of action of independent innovation on the sustainable growth of agricultural enterprises, we conducted empirical tests using data from agricultural enterprises listed on the Shanghai and Shenzhen A-shares markets from 2007 to 2021. Compared with previous research, this article makes marginal academic contributions in the following three aspects.

Firstly, this study used principal component analysis (PCA) to construct an evaluation index system for the sustainable growth of agricultural enterprises. This research method is consistent with the research on high-quality development of enterprises [53,54]. At the same time, this index system incorporates the heterogeneity of agricultural enterprises [79]. The study found that the incentive effect of independent innovation on the sustainable growth of agricultural enterprises exhibits an “inverted U-shaped” pattern. This conclusion validates the research on the effect of independent innovation in initially promoting and then weakening the growth of enterprises [80,81]. As independent innovation intensity increases, it helps to enhance the economic quality and social performance of enterprises, promoting the sustainable development of agricultural enterprises. However, when independent innovation exceeds a certain threshold, factors such as “diseconomies of scale” and “risk effects” may constrain the sustainable development of agricultural enterprises.

Secondly, we further analyzed the heterogeneous impact of independent innovation based on the property rights, scale, and life cycle differences of agricultural enterprises and found that the inverted U-shaped relationship between independent innovation and non-state-owned agricultural enterprises was more significant compared with state-owned agricultural enterprises. This conclusion verifies that independent innovation has different effects on the performance of enterprises with different property rights [32]. This paper also finds that independent innovation has a significant inverted U-shaped impact on the sustainable development of mature agricultural enterprises, but the impact is not significant on growing agricultural enterprises and declining agricultural enterprises. This also confirms that the innovation activities and economic efficiency of enterprises vary across different life cycles [82,83]. These new findings will help agricultural enterprises rationally adjust the intensity and pace of independent innovation, better define their own positioning and development direction, and jointly promote the sustainable development of the agricultural industry. In addition, this study also found that independent innovation has significant differences in the economic output of enterprises of different sizes. This finding is consistent with the research conducted by Mahirun and Kushermant [84], which further confirms that enterprises of different sizes respond differently to economic activities [85,86]. For agricultural enterprises, large agricultural enterprises are the first to reach the optimal threshold of independent innovation, while small and medium-sized agricultural enterprises are relatively lagging behind in reaching the optimal threshold of independent innovation, and the corresponding incentive effect range is also longer.

Third, this study found that digital transformation not only helps to strengthen the positive impact of independent innovation before reaching the threshold on the sustainable growth of agricultural enterprises but also helps to mitigate the negative impact of independent innovation beyond the threshold on the sustainable growth of agricultural enterprises. This conclusion confirms that the internal transformation drive plays a crucial moderating role between independent innovation and enterprise performance [33,87]. At the same time, this study found that government subsidies were conducive to the sustainable growth of agricultural enterprises in the early stages of independent innovation but prone to rent-seeking behaviors in agricultural enterprises during the later stages of independent innovation. This conclusion also confirmed that external driving forces such as government subsidies played a key regulatory role between independent innovation and enterprise operation and growth [88,89,90,91]. Overall, the main contribution of this section lies in the in-depth exploration of which one played a more significant role in the process of independent innovation for agricultural enterprises, thereby enriching the research results in the field of enterprise innovation.

It should be noted that this study has several limitations. In terms of research scope, due to time and resource constraints, the study focused only on listed agricultural enterprises in China. For a broader range of research subjects, such as unlisted agricultural enterprises in China and international agricultural enterprises, due to the difficulty in obtaining data, we have not been able to conduct in-depth discussions. Furthermore, during the research process, it was not possible to completely exclude the potential impact of external factors, such as economic environment fluctuations, the impact of the pandemic, and the conflict between Russia and Ukraine, which might increase economic uncertainties and affect the impact of independent innovation on the sustainable growth of agricultural enterprises.

## 7. Conclusions and Policy Recommendations

### 7.1. Research Conclusions

Based on the data of agricultural enterprises listed on the Shanghai and Shenzhen A-share markets from 2007 to 2021, this study constructed an index system for the sustainable growth of agricultural enterprises and empirically tests the effect and regulatory mechanism of self-innovation on the sustainable growth of agricultural enterprises. The findings reveal the following: (1) The incentive effect of self-innovation on the sustainable growth of agricultural enterprises exhibits a U-shaped nonlinear characteristic, initially positive and then negative. When the R&D investment of agricultural companies accounts for 77.85% of their operating income, the effect of promoting sustainable growth is most evident. (2) The impact of independent innovation on the sustainable growth of non-state-owned agricultural enterprises and agricultural enterprises in the mature stage is “inverted U-shaped”, and there is no significant relationship between independent innovation and the sustainable growth of state-owned agricultural enterprises and agricultural enterprises in the growth and decline stages. Compared with large-scale agricultural enterprises, small and medium-sized ones reach the threshold of independent innovation later and enjoy a longer range of incentive effects brought by independent innovation. (3) Investment in digital transformation can not only promote the positive impact on the sustainable growth of agricultural enterprises when self-innovation does not exceed the threshold but also mitigate the negative impact on the sustainable growth of agricultural enterprises when self-innovation exceeds the threshold. Government subsidies can strengthen the positive impact of the early stages of independent innovation on the sustainable growth of agricultural enterprises but cannot mitigate the negative impact of the later stages of independent innovation on the sustainable growth of agricultural enterprises. Therefore, on the path to sustainable growth of agricultural enterprises led by independent innovation, internally driven digital transformation plays a more significant moderating role than externally driven government subsidies.

### 7.2. Policy Recommendations

Based on the above research conclusions, this paper puts forward the following policy recommendations. First, measures should be tailored to different categories of agricultural enterprises to encourage independent innovation. (1) For agricultural enterprises whose independent innovation is nearing the threshold, they should not solely depend on breakthroughs in key core technologies but should focus on optimizing the governance structure, strengthening supply chain management, and enhancing market research and forecasting. At the same time, improve the enterprise risk early warning mechanism and strengthen strategic planning. (2) For agricultural enterprises whose independent innovation has just reached the threshold, optimize the structure of R&D investment, focus on these enterprises and play an early warning role. Consolidate current advantages in innovation and sustainable development, strengthen the protection and optimization of core innovation achievements. Actively fulfill social responsibilities, improve the corporate social image, and promote a virtuous circle of sustainable development. (3) For agricultural enterprises whose independent innovation has exceeded the threshold, rebalance resources, adjust resource allocation, increase investment in production and marketing, ensure innovative achievements are effectively brought to market and realize value. Strengthen cooperation and sharing, engage in innovation cooperation with other enterprises or institutions, and share innovation resources and achievements. Provide guidance on risk prevention and management, offer professional guidance on risk assessment and prevention and control, and help enterprises identify and address potential risks from excessive innovation. Provide guidance on innovation spillover, encourage agricultural enterprises to share their excess innovation capacity with other industry players, and stimulate the innovative development of the entire industry. (4) For agricultural enterprises whose independent innovation has not reached the threshold, they should strengthen their investment in research and development (R&D), increase capital and manpower inputs for R&D, pay attention to cultivating and attracting talents with innovative ability, establish innovation cooperation networks, establish cooperative relationships with other enterprises, scientific research institutions, government departments, etc., jointly carry out innovation projects, and improve their independent innovation ability.

Second, agricultural enterprises should intensify their investment in digital transformation, including technology and capital investment, to enhance their digital capabilities. Efforts should be made to address the shortcomings in basic software and high-end digital technology facilities, with a focus on deepening the integration of digital technologies, such as agricultural big data and intelligent information equipment, to fully leverage the role of digital technology in innovation efficiency and resource allocation efficiency. At the same time, government departments should formulate relevant policies to encourage agricultural enterprises and industries to realize digital transformation as soon as possible. Third, agricultural enterprises should make rational use of government subsidies. In the early stage of independent innovation, they should fully utilize government subsidies for technological research and development, talent training, and equipment procurement. In the later stage, agricultural enterprises should reduce their dependence on government subsidies and focus on improving their innovation capabilities and market competitiveness. Finally, the government should support different types of agricultural enterprises through categorized policies. State-owned agricultural enterprises, large-scale agricultural enterprises, and those in the growth and decline stages have a weaker driving force for independent innovation due to their own characteristics or financing constraints. The government should provide corresponding policy support to help them optimize the financing environment, strengthen market supervision and information services, and promote industrial integration and collaborative development.

## Figures and Tables

**Table 1:** Variable definitions.

Types of Variables	Variable Name		Symbol	Variable Definition
Sustainable growth of agricultural enterprises	Profitability	Return on total assets	X1	(total profit + financial expenses)/total assets × 100%
		Operating gross margin	X2	(operating revenue − operating cost)/operating revenue × 100%
		Net profit margin on sales	X3	Net profit/sales revenue × 100%
		Return on investment capital	X4	Investment income/invested capital × 100%
	Scale capacity	Scale of fixed assets	X5	Proportion of fixed assets in the industry
		Operating revenue scale	X6	Proportion of sales revenue in the industry
	Solvency	Quick ratio	X7	(current assets − inventory)/current liabilities × 100%
		Current ratio	X8	Current assets/current liabilities × 100%
	Growth capacity	Growth rate of total assets	X9	Growth rate of total assets over the previous year
		Growth rate of operating revenue	X10	Growth rate of operating income over the previous year
	Social performance	Social donation	X11	Contributions to society
		External guarantee	X12	External credit guarantee support
Core explanatory variable	Independent innovation		R&D	Proportion of R&D investment in operating income%
Adjusting variable	Investment intensity in digital transformation		lnDig	Logarithm of enterprise digital transformation investment
	Government subsidies		Sub	Proportion of government subsidies in operating income
Control variables	Enterprise size		Size	Logarithm of assets at the end of the year
	P/E ratio		Pe	Current market price per common share/annual earnings per common share × 100%
	Total asset turnover		Tat	Operating income/total assets closing balance × 100%

**Table 2:** Results of descriptive statistics of variables.

Variable	Mean	Std. Dev.	Min	Median	Max
SGAE	−0.004	1.623	−9.990	−0.270	9.760
R&D	0.013	0.017	0	0.006	0.145
lnDig	15.400	1.982	−1.715	15.600	19.410
Sub	0.006	0.012	0	0.003	0.162
Se	21.860	1.105	18.580	21.720	25.900
Pe	0.782	1.570	0	0.373	24.410
Tat	0.810	0.576	0.020	0.674	5.412
Soe	0.351	0.477	0	0	1
Size	0.214	0.410	0	0	1
Lifecycle	0.769	0.737	0	1	2

**Table 3:** Regression results of the main effect of R&D investment on the sustainable growth of agricultural enterprises.

Variable	(1)	(2)	(3)	(4)	(5)	(6)
R&D	4.663 ***		8.372 ***	8.726 ***	1.504 ***	30.922 **
	(3.75)		(6.19)	(3.76)	(5.38)	(2.31)
R&D^2^					−0.966 ***	−3.499 *
					(−3.24)	(−1.72)
Se		0.305 ***	0.328 ***	0.119 ***	0.331 ***	0.089 **
		(10.07)	(10.46)	(2.85)	(10.53)	(2.19)
Pe		−0.001 ***	−0.001 ***	−0.001 ***	−0.001 ***	−0.001 ***
		(−4.76)	(−4.52)	(−3.69)	(−4.40)	(−4.03)
Tat		0.334 ***	0.338 ***	0.359 ***	0.337 ***	0.362 ***
		(7.87)	(7.96)	(4.85)	(7.93)	(5.08)
Constant	0.233 *	−6.183 ***	−6.778 ***	−2.909 ***	−6.828 ***	−2.088 **
	(1.86)	(−9.50)	(−9.98)	(−3.14)	(−10.04)	(−2.30)
Observations	2451	2145	2076	1870	2076	1961
R-squared	0.502	0.577	0.588	0.049	0.589	0.040
Industry	YES	YES	YES	YES	YES	YES
Year	YES	YES	YES	YES	YES	YES
U-test					Pass	Pass
Cragg-Donald Wald F				5957.870		309.401

Note: the data in brackets are t values *** Indicates *p* < 0.01, ** indicates *p* < 0.05, * indicates *p* < 0.1, the same below.

**Table 4:** Robustness-test results.

Variable	(1)	(2)	(3)	(4)	(5)	(6)
	ROE	NPOA	ROA	Sgae (2010–2021)	No Digital Transformation	R&D/Total Assets
R&D	8.643 *	1.028 ***	1.084 ***	12.956 ***	9.221 ***	
	(1.93)	(5.31)	(5.20)	(4.46)	(5.37)	
R&D^2^	−0.666 *	−0.070 ***	−0.082 ***	−0.857 ***	−0.300 ***	
	(−1.75)	(−3.26)	(−3.73)	(−2.79)	(−5.28)	
R&D/Assets						0.309 ***
						(5.29)
R&D/Assets^2^						−0.411 ***
						(−2.95)
Controls	YES	YES	YES	YES	YES	YES
Constant	−0.593 **	−0.325 ***	−0.392 ***	−5.302 ***	−7.278 ***	−7.020 ***
	(−2.19)	(−7.20)	(−7.99)	(−7.28)	(−11.11)	(−10.49)
Observations	2076	2076	2076	1772	1535	2044
R-squared	0.014	0.180	0.213	0.580	0.564	0.587
Industry	YES	YES	YES	YES	YES	YES
Year	YES	YES	YES	YES	YES	YES

Note: the data in brackets are t values. *** Indicates *p* < 0.01, ** indicates *p* < 0.05, * indicates *p* < 0.1, the same below.

**Table 5:** Regression results for heterogeneity in the nature of property rights.

Variable	Full Sample	State-Owned Agricultural Enterprises	Non-State-Owned Agricultural Enterprises
R&D	1.504 ***	1.759	15.977 ***
	(5.38)	(0.32)	(4.44)
R&D^2^	−0.966 ***	−0.021	−1.231 ***
	(−3.24)	(−0.03)	(−3.31)
Controls	YES	YES	YES
Constant	−6.828 ***	−9.697 ***	−5.017 ***
	(−10.04)	(−12.17)	(−4.93)
Observations	2076	870	1206
R-squared	0.589	0.678	0.561
Industry	YES	YES	YES
Year	YES	YES	YES
Chow Test		12.93 ***	

Note: the data in brackets are t values. *** Indicates *p* < 0.01, the same below.

**Table 6:** Regression results of the heterogeneity of agricultural enterprise scale.

Variable	Full Sample	Large Agricultural Enterprises	Small- and Medium-Sized Agricultural Enterprises
R&D	1.504 ***	1.329 **	1.351 ***
	(5.38)	(2.52)	(3.87)
R&D^2^	−0.966 ***	−1.275 *	−0.699 *
	(−3.24)	(−1.71)	(−1.92)
Controls	YES	YES	YES
Constant	−6.828 ***	−7.380 ***	−9.439 ***
	(−10.04)	(−6.70)	(−4.34)
R-squared	0.589	1068	1008
Industry	YES	0.563	0.639
Year	YES	YES	YES
Chowtest		4.65 ***	

Note: the data in brackets are t values. *** Indicates *p* < 0.01, ** indicates *p* < 0.05, * indicates *p* < 0.1, the same below.

**Table 7:** Criteria for dividing the life cycle of agricultural enterprises.

Cash Flow Symbol	Growth Period		Mature Period			Recession Period		
	Start Up	Grow Up	Mature	Turbulence	Turbulence	Turbulence	Decline	Decline
Operating activities	negative	positive	positive	negative	positive	positive	negative	negative
Investment activities	negative	negative	negative	negative	positive	positive	positive	positive
Fundraising activities	positive	positive	negative	negative	positive	negative	positive	negative

**Table 8:** Regression results of heterogeneity in the agribusiness life cycle.

Variable	Full Sample	Growth Period	Mature Period	Recession Period
R&D	1.504 ***	0.150 ***	0.400 ***	0.520 **
	(5.38)	(2.68)	(3.91)	(2.41)
R&D^2^	−0.966 ***	−1.106	−6.585 ***	−8.522
	(−3.24)	(−0.95)	(−2.73)	(−1.44)
Controls	YES	YES	YES	YES
Constant	−6.828 ***	−2.631 ***	−8.986 ***	−7.546 ***
	(−10.04)	(−3.09)	(−10.63)	(−3.25)
Observations	2076	806	813	359
R-squared	0.589	0.688	0.608	0.504
Industry	YES	YES	YES	YES
Year	YES	YES	YES	YES
Chowtest		11.10 ***		

Note: the data in brackets are t values. *** Indicates *p* < 0.01, ** indicates *p* < 0.05, the same below.

**Table 9:** The moderating effect of investment intensity in digital transformation on self-innovation and the sustainable growth of agricultural enterprises.

	(1)	(2)	(3)	(4)
VARIABLES	SGAE	SGAE	SGAE	SGAE
R&D	6.010 ***	7.887 ***	0.160 ***	0.035 **
	(5.78)	(4.14)	(5.33)	(2.43)
R&D^2^	−1.055 ***	−3.812 **	−0.982 ***	−0.004 *
	(−3.40)	(−2.22)	(−3.28)	(−1.65)
lnDig	0.031 *	0.039 **		
	(1.71)	(2.02)		
R&D × lnDig	3.000 ***	−4.247 ***		
	(4.73)	(−3.36)		
R&D^2^ × lnDig		1.845 **		
		(2.21)		
Sub			2.512 **	−1.877 ***
			(2.01)	(−2.95)
R&D × Sub			1.336 ***	1.657 *
			(6.2)	(1.90)
R&D^2^ × Sub				−0.325 *
				(−1.80)
Controls	YES	YES	YES	YES
Constant	−8.283 ***	−8.383 ***	−6.753 ***	−0.842 ***
	(−11.91)	(−12.03)	(−9.84)	(−6.82)
Observations	1689	1689	2076	2044
R-squared	0.604	0.605	0.590	0.800
Industry	YES	YES	YES	YES
Year	YES	YES	YES	YES
U-test	Pass	Pass	Pass	Pass

Note: the data in brackets are t values. *** Indicates *p* < 0.01, ** indicates *p* < 0.05, * indicates *p* < 0.1, the same below.

## Data Availability

The original contributions presented in the study are included in the article, further inquiries can be directed to the corresponding author.
